# Laparoscopy-assisted total colectomy for progressive megacolon due to intestinal ganglioneuromatosis in a young adult with multiple endocrine neoplasia type 2B: a case report

**DOI:** 10.1016/j.ijscr.2025.112028

**Published:** 2025-10-06

**Authors:** Yoshifumi Shimada, Akio Matsumoto, Kaori Takamura, Takashi Kobayashi, Yoshiaki Kinoshita, Toshifumi Wakai

**Affiliations:** a Division of Digestive and General Surgery, Niigata University Graduate School of Medical and Dental Sciences, Niigata, Japan; b Department of Genome Medicine, Niigata University Medical and Dental Hospital, Niigata, Japan; c Division of Molecular and Diagnostic Pathology, Niigata University Graduate School of Medical and Dental Sciences, Niigata, Japan; d Department of Pediatric Surgery, Niigata University Graduate School of Medical and Dental Sciences, Niigata, Japan

**Keywords:** Laparoscopic surgery, Adolescent and young adult, Multiple endocrine neoplasia type 2B, Megacolon, Intestinal ganglioneuromatosis

## Abstract

**Introduction:**

Multiple endocrine neoplasia type 2B (MEN2B) is a rare hereditary syndrome requiring multidisciplinary management from pediatric to adult care. Gastrointestinal manifestations, particularly megacolon due to intestinal ganglioneuromatosis, pose significant challenges during the adolescent and young adult (AYA) transition period. Here, we report the first case of laparoscopy-assisted total colectomy for MEN2B-associated megacolon caused by intestinal ganglioneuromatosis.

**Presentation of case:**

At age 13, he was evaluated for oral cavity masses, and was diagnosed with mucosal neuromas of his tongue, raising suspicion for MEN2B. Genetic testing confirmed a *RET* M918T variant, and he subsequently underwent total thyroidectomy for medullary thyroid carcinoma. At age 16, he underwent partial resection of the descending colon for recurrent diverticulitis caused by intestinal ganglioneuromatosis. Subsequently, he experienced alternating bouts of constipation and diarrhea with progressive colonic dilation. At ages 26 and 27, he was hospitalized for bowel obstruction due to progressive megacolon. After conservative treatment, he underwent laparoscopy-assisted total colectomy with ileorectal anastomosis.

**Discussion:**

Our case demonstrates that laparoscopy-assisted total colectomy is both technically feasible and effective for treating megacolon in patients with MEN2B. This approach may be particularly beneficial for these patients, who often require multiple surgeries throughout their lives due to the syndromic nature of their disease. Regular monitoring for recurrent gastrointestinal symptoms will be essential for early detection of any future involvement of the remaining intestinal tract.

**Conclusion:**

Laparoscopic surgery represents an effective, minimally invasive approach for managing progressive megacolon in AYA patients with MEN2B, offering advantages for long-term syndrome management.

## Highlights

• Laparoscopic surgery offers effective megacolon management in adolescent and young adult multiple endocrine neoplasia type 2B cases
• Laparoscopic surgery in patients with syndromes who require lifelong multiple surgeries
• First reported laparoscopy-assisted total colectomy for multiple endocrine neoplasia type 2B-associated megacolon

## 1 Introduction

Multiple endocrine neoplasia type 2B (MEN2B) is a rare autosomal dominant syndrome with an estimated prevalence ranging from 1 in 350,000 to 1 in 700,000 live births [1]. MEN2B accounts for less than 5 % of all MEN2 cases, making it one of the rarest hereditary endocrine cancer syndromes [2]. MEN2B is characterized by medullary thyroid carcinoma (MTC), pheochromocytoma, intestinal ganglioneuromatosis, and other characteristic features, such as mucosal neuroma, a peculiar face with lip thickening, and Marfan-like appearance [3]. MTC occurs in 100 % of patients with MEN2B and presents as the most aggressive of the MEN2B phenotypes. MEN2B typically presents with MTC at an early age, followed by various manifestations including pheochromocytoma and gastrointestinal symptoms, such as constipation and abdominal bloating [3]. Early diagnosis and potentially life-saving thyroidectomy are crucial in MEN2B management. After successful thyroidectomy, clinical management shifts to monitoring for pheochromocytoma (occurring in approximately 40 %–50 % of patients) and controlling gastrointestinal symptoms [[3], [4], [5], [6]]. Gastrointestinal manifestations, particularly megacolon due to intestinal ganglioneuromatosis, pose unique management challenges during the adolescent and young adult (AYA) transition period. Here, we report the first case of laparoscopy-assisted total colectomy for MEN2B-associated megacolon caused by intestinal ganglioneuromatosis.

## 2 Case report

**Fig. 1:**
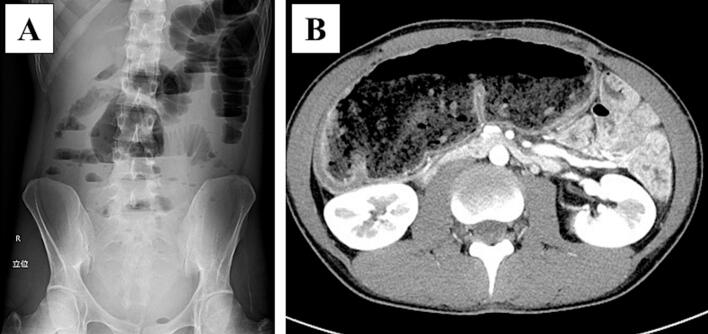
Preoperative findings of total colectomy. A Preoperative abdominal X-ray showed niveau formation. B Preoperative abdominal CT scan showed significant dilation of cecum to transverse colon.

**Fig. 2:**
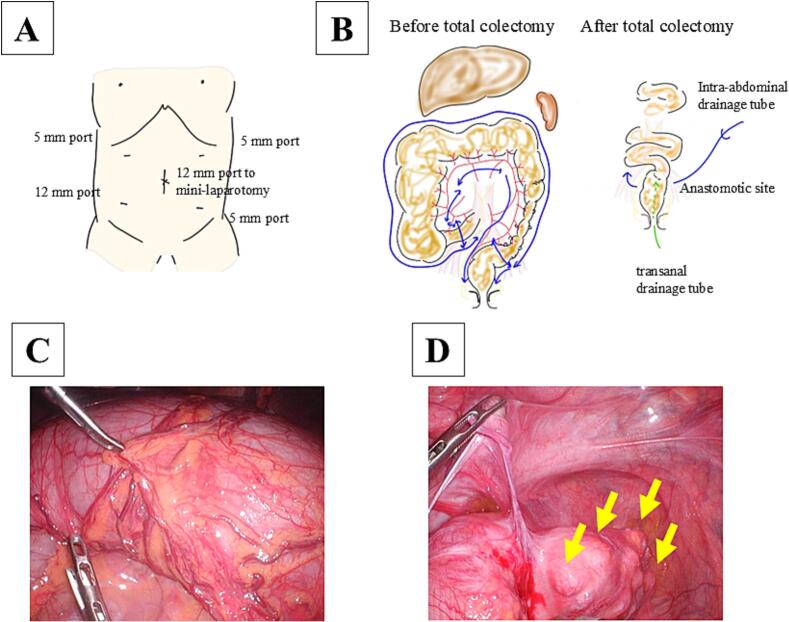
Operative findings of total colectomy. A Port placement. B Total colectomy with ileorectal anastomosis. C Megacolon of transverse colon. D Multiple diverticula of sigmoid colon with wall thickening and stenotic changes (arrows), consistent with chronic diverticulitis following previous partial colectomy.

**Fig. 3:**
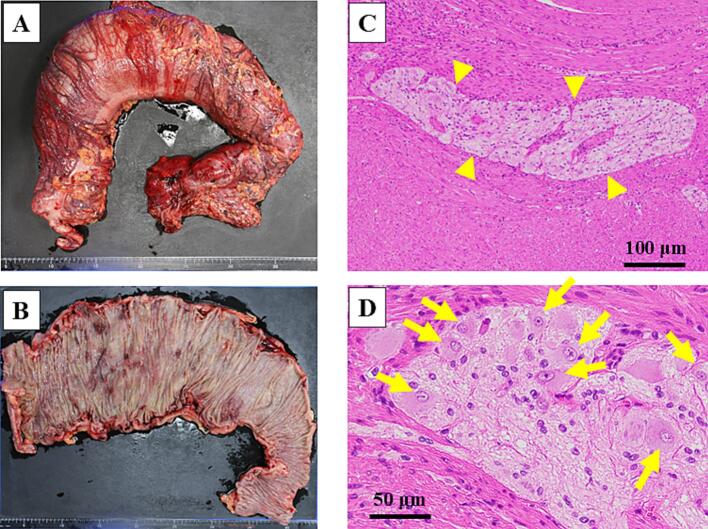
Pathological findings of the resected specimen. A, B Macroscopic findings show significant dilatation with hypertrophy of the cecum to the transverse colon. C, D Microscopic findings show intestinal ganglioneuromatosis characterized by thick nerve bundles (arrowheads) and proliferation of ganglion cells (arrows) frequently observed in the muscular layers.

This case report has been reported in line with the SCARE (Surgical CAse REport) criteria [7]. A 27-year-old man was referred to our hospital for megacolon and recurrent bowel obstructions. He has no family history of MEN2B. The patient's clinical history is summarized in Table 1. At age 13, he was evaluated for oral cavity masses, and was diagnosed with mucosal neuromas of his tongue, raising suspicion for MEN2B. Genetic testing confirmed a *RET* M918T variant, and he subsequently underwent total thyroidectomy with neck lymph node dissection for MTC. There has been no recurrence of MTC to date. He undergoes regular surveillance for pheochromocytoma, which has remained negative throughout the follow-up period. At age 16, he underwent partial resection of the descending colon through a small laparotomy incision for recurrent diverticulitis. Histopathological examination of the resected specimen revealed findings compatible with intestinal ganglioneuromatosis. Subsequently, he developed recurrent episodes of sigmoid diverticulitis requiring multiple hospitalizations and antibiotic therapy over the subsequent 10-year period (ages 16–26). He experienced progressive abdominal bloating and alternating bouts of constipation and diarrhea, with imaging revealing progressive colonic dilation and multiple diverticula formation in the sigmoid colon. Conservative management became increasingly ineffective over time. At ages 26 and 27, he was hospitalized for recurrent bowel obstructions requiring conservative treatment. Abdominal X-ray detected an air-fluid level, and computed tomography showed significant colonic dilation (Fig. 1). After conservative treatment with an ileus tube, he was referred to our hospital for treatment of recurrent bowel obstruction. Considering his clinical history of MEN2B, we attributed the megacolon and recurrent bowel obstructions to intestinal ganglioneuromatosis. Imaging revealed a significantly dilated colon from the cecum to transverse colon and multiple diverticula from the sigmoid colon to rectosigmoid, leading to an indication for total colectomy. Preoperative colonoscopy revealed no significant abnormalities in the rectum, with ganglioneuromatosis changes primarily affecting the colon. The extent of rectal dissection was determined based on both endoscopic findings and intraoperative assessment of bowel wall thickness, with the anastomosis planned at a level where normal rectal wall characteristics were confirmed. Since the small bowel dilation had improved by the time of surgery due to decompression with an ileus tube, we considered that laparoscopic visualization would be possible. He underwent laparoscopy-assisted total colectomy with ileorectal anastomosis (Fig. 2). Under laparoscopic assistance, we resected the dilated cecum to transverse colon and the descending colon to rectosigmoid with diverticula. The ileorectal anastomosis was performed using a double-stapling technique. The anastomosis was located 12 cm from the anal verge. The operating time was 211 min, and intraoperative bleeding was minimal. There were no intraoperative or postoperative complications, and he was discharged 13 days postoperatively. Abdominal bloating and constipation have dramatically improved after the operation. The macroscopic findings of the resected specimen showed a dilated cecum to transverse colon and diverticula of the sigmoid colon to rectum (Fig. 3A, B). Microscopic findings showed thick nerve bundles and proliferation of ganglion cells in the submucosal and muscular layers, consistent with intestinal ganglioneuromatosis (Fig. 3C, D).

**Table 1:** Summary of the patient's clinical course and surgical management.

Age	Clinical event	Key findings	Intervention
13	Oral mucosal neuromas; suspicion of MEN2B	Genetic testing; *RET* M918T	Total thyroidectomy with LN dissection for MTC
16	Recurrent diverticulitis of descending colon	Localized diverticulitis	Partial resection of descending colon (small laparotomy)
16–26	Recurrent diverticulitis of sigmoid colon	Localized diverticulitis	Conservative management with antibiotics
26–27	Recurrent bowel obstruction	Significant colonic dilation	Conservative management with ileus tube
27	Progressive megacolon, recurrent obstruction	Marked colonic dilation and multiple diverticula (sigmoid to rectosigmoid)	Laparoscopy-assisted total colectomy with ileorectal anastomosis

*MEN2B* multiple endocrine neoplasia type 2B, *LN* lymph node, *MTC* medullary thyroid carcinoma.

## 3 Discussion

Gastrointestinal symptoms have been reported to occur in approximately 90 % of patients with MEN2B, and these symptoms often begin in the first months of life [4]. Constipation is the most common gastrointestinal symptom, and megacolon and secondary diverticulosis are common gastrointestinal manifestations of MEN2B [4]. Intestinal ganglioneuromatosis, which is pathologically defined as thickening of the myenteric plexi and hypertrophy of ganglion cells [5], occurs in approximately 40 % of patients with MEN2B [6]. However, megacolon develops in only a subset of these patients. Studies have reported that megacolon occurs in approximately 31 % to 63 % of patients with MEN2B [5,8]. A Mayo Clinic study reported that among 11 patients with MEN2B, 7 (63 %) had megacolon [5], while an earlier study estimated the prevalence at 31 % [8]. These ganglioneuromas can be found throughout the gastrointestinal tract and lead to loss of normal bowel tone, distention, segmental dilation, and ultimately megacolon [5,9,10]. Intestinal ganglioneuromatosis is the predominant cause of gastrointestinal symptoms and manifestations in patients with MEN2B.

Megacolon is defined as an abnormal dilation of the colon, typically diagnosed when the colonic diameter exceeds standard measurements: greater than 12 cm in the cecum, 8 cm in the ascending colon, or 6.5 cm in the rectosigmoid region [6]. In our patient, the dilated ascending colon measured 8.5 cm in diameter, confirming the diagnosis of megacolon. The differential diagnosis of megacolon includes several conditions, such as toxic megacolon associated with inflammatory bowel disease or infections, Hirschsprung's disease with congenital absence of ganglion cells, acquired megacolon due to medication, and intestinal pseudo-obstruction [6]. MEN2B represents a rare endocrine cause of megacolon, with intestinal ganglioneuromatosis as the primary pathological mechanism [[4], [5], [6],[8], [9], [10], [11], [12]].

Total colectomy is sometimes required to treat gastrointestinal symptoms due to megacolon in patients with MEN2B. Several case reports describe patients with MEN2B who underwent total colectomy with permanent colostomy or ileorectal anastomosis [[4], [5], [6],[10], [11], [12]]. Typically, in patients with MEN2B, open surgery has been selected for total colectomy for megacolon [6,12]. Here, we describe a patient with MEN2B reporting gastrointestinal symptoms since childhood, who underwent laparoscopic surgery for progressive megacolon. To the best of our knowledge, this is the first report of laparoscopy-assisted total colectomy with ileorectal anastomosis for megacolon in a patient with MEN2B. A literature search was performed using PubMed (January 1990–May 2025) with the keywords “MEN2B,” “megacolon,” and “laparoscopic surgery.” No previous cases of laparoscopic colectomy for MEN2B-associated megacolon were identified.

In our patient, earlier total colectomy was deferred because his symptoms were initially manageable with conservative measures and because partial colectomy at age 16 temporarily alleviated recurrent diverticulitis. During adolescence and early adulthood, major surgery was avoided in an attempt to preserve bowel function and quality of life. However, progressive colonic dilatation with recurrent episodes of bowel obstruction ultimately necessitated total colectomy at age 27. This highlights the delicate balance between avoiding overtreatment during adolescence and preventing severe complications in the young adult period.

The timing of surgery in AYA with MEN2B remains challenging. Although conservative management may be sufficient in the early stages, colectomy should be considered earlier in patients who exhibit progressive colonic dilatation, recurrent obstruction, or impaired quality of life despite nonoperative management. Based on our experience, potential criteria for considering total colectomy during the AYA transition include radiological evidence of marked colonic dilatation (e.g., diameter exceeding 8 cm in the ascending colon), repeated hospitalizations for obstruction, or failure of prior segmental resection. Such decisions require careful multidisciplinary evaluation to balance the preservation of bowel function with the long-term benefits of preventing progression to frank megacolon.

Management options of megacolon range from conservative treatments to surgical interventions, depending on the underlying cause and severity. In patients with MEN2B, intestinal ganglioneuromatosis often leads to chronic constipation that can progress to megacolon that is resistant to conservative management [[4], [5], [6],[9], [10], [11], [12]]. The choice between open or laparoscopic approaches should be considered individually based on patient characteristics and disease severity. Factors favoring a laparoscopic approach include adequate bowel decompression prior to surgery, stable patient condition, and surgeon expertise in minimally-invasive techniques. In our case, successful preoperative decompression created optimal conditions for a laparoscopic approach.

Minimally-invasive surgical approaches offer several advantages over conventional open surgery, including reduced postoperative pain, shorter hospital stays, faster recovery, and improved cosmetic outcomes. These benefits have also led to the increasing adoption of laparoscopic techniques for patients with hereditary tumor syndromes. For patients with MEN2B specifically, laparoscopic adrenalectomy has already become the standard approach for pheochromocytoma management [13]; however, laparoscopic treatment of MEN2B-associated gastrointestinal complications has been rarely reported in the literature. To date, only two cases of open colectomy for MEN2B-associated megacolon have been reported, both with uneventful intraoperative and postoperative courses [6,12]. In contrast, our laparoscopic approach provided adequate visualization, safe vessel control, and effective resection without intraoperative complications. This case demonstrates that laparoscopy-assisted total colectomy is both technically feasible and effective for treating megacolon in patients with MEN2B. Moreover, this minimally invasive approach may be particularly beneficial for these patients, who often require multiple abdominal surgeries throughout their lifetime due to the syndromic nature of the disease.

While intestinal ganglioneuromatosis in MEN2B can theoretically affect any part of the gastrointestinal tract, including the small intestine and rectum, the predominant involvement is typically in the colon [4,8]. In our patient, preoperative evaluation including colonoscopy and intraoperative assessment revealed that ganglioneuromatosis changes were primarily limited to the colon, with the rectum appearing relatively spared. However, long-term surveillance remains important as progression of ganglioneuromatosis to involve the remaining intestinal tract. Regular monitoring for recurrent gastrointestinal symptoms will be essential for early detection of any future involvement of the remaining intestinal tract.

Regarding the timing of surgical intervention, our patient developed recurrent diverticulitis in the sigmoid colon after the initial partial colectomy at age 16. The progressive nature of intestinal ganglioneuromatosis led to worsening megacolon over the subsequent decade, with multiple hospitalizations for bowel obstruction at ages 26 and 27. While earlier total colectomy might have been considered, the decision was made to pursue conservative management initially due to the patient's young age and the hope that symptoms might be manageable with medical therapy. However, the recurrent obstructions and deteriorating quality of life ultimately necessitated definitive surgical intervention. This case highlights the challenge of timing surgical intervention in young patients with progressive MEN2B-associated gastrointestinal complications.

## 4 Conclusion

In conclusion, this case illustrates that laparoscopy-assisted total colectomy is a feasible and effective option for MEN2B-associated megacolon. Minimally invasive surgery may be particularly advantageous for these patients, who often require multiple abdominal procedures during their lifetime due to the syndromic nature of the disease.

## Author contribution

**Yoshifumi Shimada:** Writing – review & editing, Writing – original draft, Conceptualization. **Akio Matsumoto:** Writing – review & editing. **Kaori Takamura:** Writing – review & editing. **Takashi Kobayashi:** Writing – review & editing. **Yoshiaki Kinoshita:** Writing – review & editing. **Toshifumi Wakai:** Writing – review & editing, Supervision.

## Consent

Informed consent was obtained from the patient.

## Ethical approval

The Ethics Committee of Niigata University approved the study protocol (approval number: 2018-0137).

## Guarantor

Yoshifumi Shimada.

## Research registration number

Not applicable.

## Declaration of Generative AI and AI-assisted technologies in the writing process

During the preparation of this work the author used generative AI tools (Claude, Anthropic; ChatGPT, OpenAI) in order to assist in structuring and improving the clarity of selected portions based on author-provided concepts and keywords. After using this tool, the author reviewed and edited the content as needed and take full responsibility for the content of the publication.

## Funding

None.

## Conflict of interest statement

The authors declare that they have no current financial arrangement or affiliation with any organization which may have a direct influence on their work.
